# Environmental quality alters female costs and benefits of evolving under enforced monogamy

**DOI:** 10.1186/1471-2148-14-21

**Published:** 2014-02-05

**Authors:** Vera M Grazer, Marco Demont, Łukasz Michalczyk, Matthew JG Gage, Oliver Y Martin

**Affiliations:** 1 ETH Zürich, Institute of Integrative Biology, D-USYS, Universitätsstrasse 16, CH-8092 Zürich, Switzerland; 2 Department of Entomology, Institute of Zoology, Jagiellonian University, Gronostajowa 9, 30-387 Kraków, Poland; 3 School of Biological Sciences, University of East Anglia, Norwich NR4 7TJ, United Kingdom

**Keywords:** Sexual conflict, *Tribolium*, Polyandry, Monogamy, Mating costs, Reproductive success

## Abstract

**Background:**

Currently many habitats suffer from quality loss due to environmental change. As a consequence, evolutionary trajectories might shift due to environmental effects and potentially increase extinction risk of resident populations. Nevertheless, environmental variation has rarely been incorporated in studies of sexual selection and sexual conflict, although local environments and individuals’ condition undoubtedly influence costs and benefits. Here, we utilise polyandrous and monogamous selection lines of flour beetles, which evolved in presence or absence of sexual selection for 39 generations. We specifically investigated effects of low *vs.* standard food quality (i.e. stressful *vs*. benign environments) on reproductive success of cross pairs between beetles from the contrasting female and male selection histories to assess gender effects driving fitness.

**Results:**

We found a clear interaction of food quality, male selection history and female selection history. Monogamous females generally performed more poorly than polyandrous counterparts, but reproductive success was shaped by selection history of their mates and environmental quality. When monogamous females were paired with polyandrous males in the standard benign environment, females seemed to incur costs, possibly due to sexual conflict. In contrast, in the novel stressful environment, monogamous females profited from mating with polyandrous males, indicating benefits of sexual selection outweigh costs.

**Conclusions:**

Our findings suggest that costs and benefits of sexually selected adaptations in both sexes can be profoundly altered by environmental quality. With regard to understanding possible impacts of environmental change, our results further show that the ecology of mating systems and associated selection pressures should be considered in greater detail.

## Background

Rather little is known about how benefits of polyandry [1-5] are affected by different environmental conditions. Environmental change, in particular human-induced modifications in the landscape, can expose populations to suboptimal breeding conditions. For example, landscape fragmentation results in disconnected small habitats, which are often of poor quality as reviewed by Ewers and Didham [6]. Among remnant patches migration can be constrained [7-9] and as a consequence, foraging success within poor quality habitats may be low, leading to decreased reproductive output of the confined populations [6]. Successful reproduction and hence population persistence are particularly under threat in changing environments, as stochastic environmental events [10] or inbreeding [11-13] can pose severe extinction risks. A study by Martin et al. [14] suggests that benefits of polyandry due to sexual selection can depend strongly on the availability of multiple mates. However, land-use changes could degrade habitat quality and decrease local population sizes such that multiple partners can no longer be found [15]. In addition, environmental changes can also affect dispersal rates, temporarily leading to sex-biased population structures [15,16].

Changing environments could alter mating regimes and the strength of sexual selection via shifts in operational sex ratios. Decreased polyandry and weaker sexual selection could prove costly, because sexual selection has been shown to be beneficial via acting against disease [17,18], deleterious mutations [19-21], inbreeding [22-24] or selfish genetic elements [4,25]. A model using data from bird introductions to New Zealand suggested that monogamy, where sexual selection intensity is greatly reduced compared to polygamy, can increase extinction risk particularly in short lived species [26]. Sexual selection can also be a handicap, e.g. for population establishment in a novel habitat, as shown by Sorci et al. [27]. These authors proposed that polygamous organisms might pay higher energetic costs for reproduction, which then decreases resources available for maintenance or immune defence. In particular, the expression of sexually selected traits, elaborate mating behaviours and elevated investments in gametes might be costly [28]. Relaxed sexual selection could free organisms from such costs. Furthermore, there is evidence that factors such as water pollution and extreme climates can directly affect sexual selection [29-32].

The mate choice and competition mechanisms underlying sexual selection are expected to be condition dependent, and Candolin and Heuschele [33] as well as Ingleby et al. [34] predict varying reproductive outcomes across different environments. Specifically, individual condition, phenotypic plasticity and genotype-by-environment interactions can change the expression of sexually selected signals and their perception [33,35-38]. Tanaka [39] has theoretically shown that costs of sexually selected signals and their genetically linked perception might increase dramatically when environmental change is very rapid. At the population level, this might increase extinction risk [40]. Individual condition could also influence the outcome of sexual conflict, because it is likely that manipulation and resistance adaptations are costly. This could be the case for traits involved in precopulatory conflict, such as premating struggles [41,42], as well as for traits involved in postcopulatory conflict, such as manipulative seminal substances [43]. Considering these examples, it is conceivable that environmental change will affect costs and benefits of whole sets of traits as well as the underlying sexual selection and sexual conflict mechanisms. Ewers & Didham [6] suggested using controlled lab experiments to investigate mechanisms in response to environmental changes such as habitat fragmentation or deterioration.

In the present study, different potential consequences of environmental change were approached using long-term experimental evolution [40] lines of the naturally polygamous species *Tribolium castaneum.* Specifically, we used the selection lines from the polyandrous and monogamous selection regimes described in Demont et al. [44], where intensity of sexual selection was manipulated to steer evolutionary trajectories in different directions. By enforcing monogamy with random mate assignment, adaptations due to sexual selection and sexual conflict including their potential costs and benefits were shut down. Enforced monogamy can potentially simulate a situation under low population density where finding multiple mates is impeded and mating occurs with the first mate encountered [15]. In the other subpopulations, we allowed polyandry by giving single females access to five males simultaneously. Thereby, polyandry allows increased pre- and postcopulatory mechanisms of mate choice and competition to act, selecting for adaptations in both sexes with associated fitness costs and benefits [44,45]. Here we used these polyandrous and monogamous selection lines to investigate effects of poor food quality *vs.* standard food quality, which could represent decreased resource availability due to habitat deterioration, on costs and benefits of adaptations and/or loss of adaptations to the contrasting selection regimes. Cahill et al. emphasise that food availability is an important factor to consider with regard to extinction risk [46]. For our model organism the environment and individual condition are intimately linked, because flour beetles live in and on their food source. It has been shown that access to food [47], food quality [48], population size [49] and a rapid temperature change [50] all have the potential to alter sexual selection pressures in this species. We crossed males and females between lines of the same and the opposite selection regime in order to compare the influence of a foreign male with monogamous background *vs.* a foreign male with polyandrous background on monogamous and polyandrous females. Moreover, we replicated all crosses in a standard and a low food quality treatment (i.e. benign *vs*. stressful environments). Combined this allowed us to investigate the impact of both male and female selection histories and their interaction on reproductive success, and how these processes are altered by environmental quality.

## Methods

### Experimental evolution

We used three polyandrous (**P**_**A**_, **P**_**B**_, **P**_**C**_) and three monogamous (**M**_**A**_, **M**_**B**_, **M**_**C**_) selection lines of *Tribolium castaneum* flour beetles (see [44] for details) after 39 non-overlapping generations of selection*.***M**-lines evolved in absence of sexual selection and conflict, as one mate was randomly assigned to single females (i.e. 20 pairs; estimated *N*_*e*_ = 40), whereas in **P**-lines sexual selection and conflict were present as single females were housed together with five males (i.e. 12 groups; estimated *N*_*e*_ = 40). Beetles were able to reproduce for seven to ten days in separate 5 cm Petri dishes with ca. 10 g flour-yeast mix (organic white wheat flour with 10% brewers yeast). Then the adults were removed and offspring were pooled within lines with ample flour-yeast mix to avoid crowding. Pupae were collected randomly from these pools to separate males and females for the next generation (started when beetles were at least ten days old). Our standard rearing temperature was 30°C. *T. castaneum* is commonly fed with flour supplemented with yeast, because white wheat flour does not provide sufficient amounts of certain necessary amino acids, and productivity is greatly reduced without yeast [51].

### Reproductive success (RS) of between line crosses in two different environments

**Figure 1:**
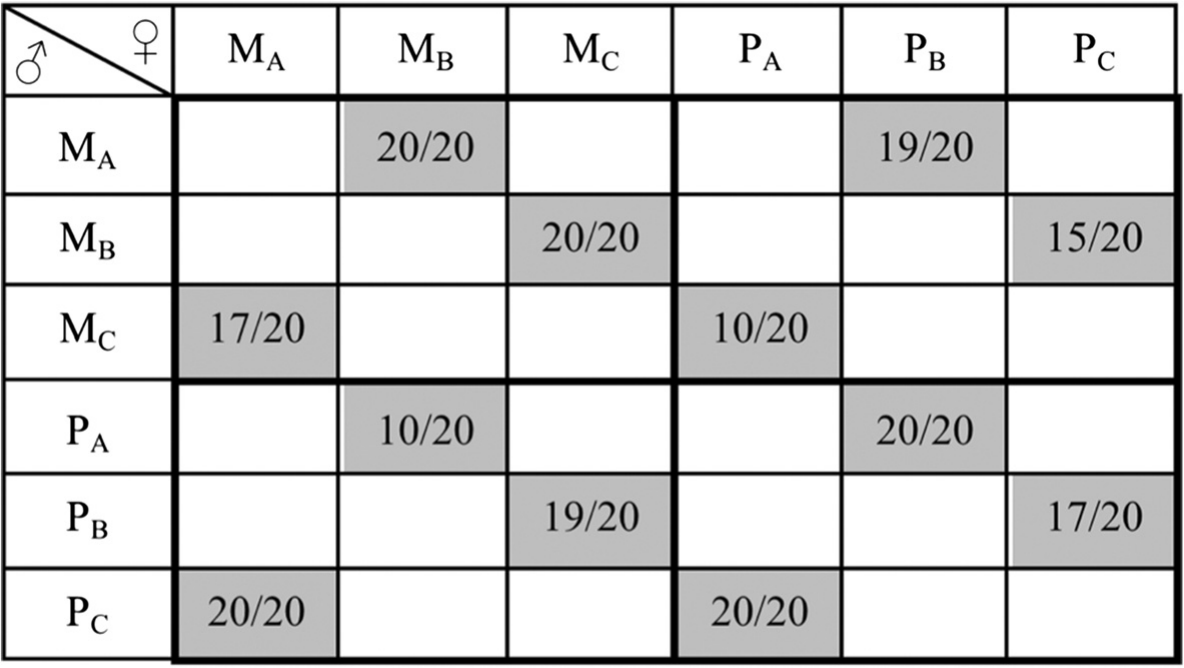
**Performed crosses.** The numbers in the shaded boxes indicate the pairs per cross among selection lines: left = low food quality treatment (1% yeast; stressful environment), right = standard food quality treatment (10% yeast; benign environment).

To ensure virginity, all animals were separated by sex as pupae (generation 39). Single sex groups (ca. 20 beetles) had access to flour-mix ad libitum (10 g per 5 cm Petri dish). Equal numbers of pupae were placed in one of two different food treatments. We used flour-mix with 10% yeast as our standard quality food treatment (= benign environment). In contrast, low quality food contained only 1% yeast (= stressful environment). After reaching maturity (all beetles >10 d post emergence) we crossed **M**- and **P**-lines within and between sexual selection regimes, without crossing individuals from the same line. Only the ♂ × ♀ crosses indicated in Figure 1 were performed, so that each line was only used once per cross type. For example, line M_A_ females were used in a single M × M cross and a single M × P cross, rather than being used in all possible combinations. Sample sizes are shown in Figure 1. Pairs were used in order to allow us to generate comparable individual fitness measures across treatment combinations. The pairs were allowed to mate and lay eggs in Petri dishes (5 cm) containing ca. 10 g flour-yeast mix (1% or 10% yeast) for 14 days. To avoid crowding of the larvae, the pairs were transferred to a new Petri dish with fresh flour-yeast mix for a further 14 days (again ca. 10 g with the same yeast content as in the first period according to treatment). Adults were removed after four weeks in total, and RS was measured as the total number of offspring produced over this period. We deliberately chose a period of four weeks as a compromise between a time window close to the selection period (seven to ten days) and the natural average life span of this species (several months). Of a total of 447 pairs in the experiment, eleven pairs had no offspring, which were distributed across all crosses and treatments.

### Data analysis

We analysed the influence of selection history of males and females (**M***vs.***P**) and food quality (low *vs.* standard) on RS with a linear mixed model using the *lme* function (nlme package) in R (version 2.13.0, R Development Core Team 2011). The explanatory variables included male selection history, female selection history, food quality and all possible interactions. Furthermore, we included a random factor cross (three population crosses for each ♂ × ♀-type (**M × M, M × P, P × M** or **P × P**), i.e. 12 different crosses, see Figure 1). The analysis of RS was performed on the full dataset including all pairs and on a reduced dataset excluding pairs, which did not produce offspring. Qualitatively these alternative analyses were equal, as the same factors and interactions were significant, and significance increased when the eleven pairs with no offspring were excluded. Thus, in the interests of being conservative and, because a lack of offspring may be biologically meaningful, results based on all pairs are shown. The residuals of the presented model were inspected visually and were normally distributed.

## Results

**Figure 2:**
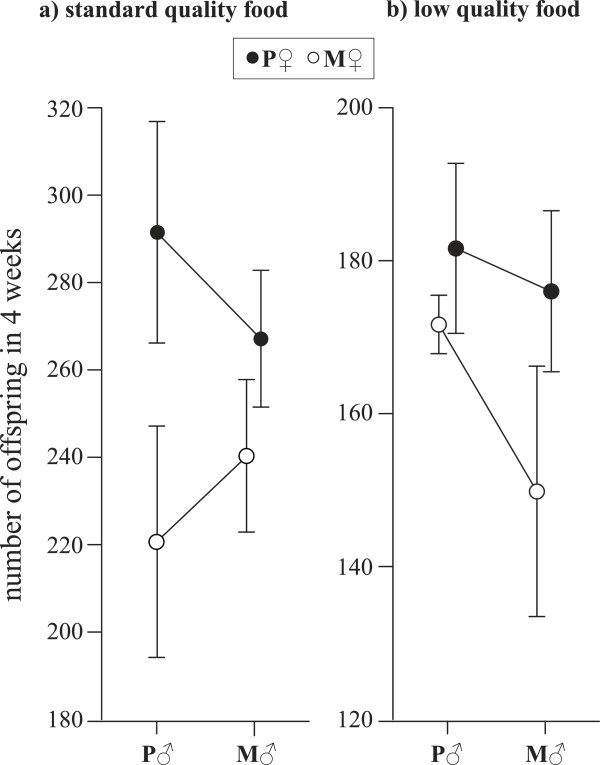
**Reproductive success.** Beetles were crossed (1♂ × 1♀) between monogamous (**M**) and polyandrous (**P**) selection lines in two contrasting (benign *vs.* stressful) environments: **a)** standard quality food = white flour with 10% yeast and **b)** low quality food = white flour with 1% yeast. Note the different scales. Each data point represents the mean ± 1 SE of three cross types (n = 10–20 pairs per cross type).

We found a significant three-way interaction between male selection history, female selection history and food quality on RS (Table 1). This three-way interaction indicates that the two-way interaction between male and female selection history differs depending on food quality. Specifically, on standard food, mating with **P**-males only resulted in higher RS if the female also derived from the polyandrous selection regime. If the female had evolved under monogamy, RS was similar (Figure 2a). In contrast, on low quality food, all cross types (♂ × ♀) where a **P**-individual was involved (i.e. **M** × **P**, **P** × **M**, **P** × **P**) had higher RS, whereas **M** × **M** crosses had the lowest offspring production on average (Figure 2b). There was a significant interaction between female selection history and food quality and a marginally non-significant main effect of female selection history (Table 1). There was a generally negative fitness effect of **M**-females (see Figure 2), except when **M**-females were paired with **P**-males in the stressful low quality food environment (Figure 2a). So, the key difference when comparing across the two environments is the cross between **M**-females and **P**-males. The highly significant effect of food quality shows that beetles, regardless of their selection history and cross type, exposed to low food quality produced markedly fewer offspring than with standard food quality (note the different scales of Figure 2a *vs.*2b).

**Table 1:** Results of the linear mixed model for reproductive success

	***ndf***	***ddf***	***F***	***p***
Food quality	1	431	168.78	**<.0001**
Female selection history	1	8	5.10	0.054
Male selection history	1	8	0.36	0.565
Food quality × female selection history	1	431	6.56	**0.011**
Food quality × male selection history	1	431	1.53	0.216
Female sel. hist. × male sel. hist.	1	8	0.33	0.579
Food quality × female sel. hist. × male sel. hist.	1	431	4.96	**0.026**

Significant effects at the 5% level are indicated in bold. Cross was used as a random factor (12 population crosses, three for each of the four ♂ × ♀-types). Variance components for the random factor: 4810.33, S.D. 69.36.

## Discussion

As predicted by conceptual work [30,34,36,52], offspring production of beetles with contrasting sexual selection histories was greatly affected by environmental quality. **M**-females generally showed inferior fitness, yet profited from mating with **P**-males in the stressful low quality food environment. Prior studies on *T. castaneum* showed that access to food is a crucial factor for sperm production and oviposition [47,48]. Not surprisingly, over four weeks of oviposition, females produced on average only 168 offspring on flour with 1% yeast compared with 254 offspring on flour with 10% yeast. This large quantitative difference indicates that beetles in the two environmental treatments had very different amounts of energy available for reproduction. Strikingly, though, the contrasting environments further revealed qualitatively different male–female interactions. This indicates that the available energy was invested differently among crosses, potentially due to adaptations or loss of adaptations in response to evolving under monogamy or polyandry. An earlier study, which specifically investigated responses to these divergent selection regimes, found adaptations to polyandry in both sexes [44]. Polyandrous males evolved to be faster than monogamous males at initiating copulation when in competition and polyandrous females seemed to be choosier than monogamous females when given a choice of males. In addition, these behaviours likely contributed to the main finding that polyandrous individuals had higher reproductive success than monogamous individuals when multiple males were present. This suggests that manipulating intensity of sexual selection has indeed led to different evolutionary trajectories potentially affecting not only precopulatory but also postcopulatory reproductive traits (see also [45]). In addition to the results of the earlier study [44], which revealed that polyandrous individuals depended on the mating setting with multiple mates to benefit from adaptations to this regime, the contrasting environments applied here indicate that food quality can also determine benefits of polyandry.

In the previous experiments with these selection lines, where males and females were crossed with tester beetles from a stock population, there were no differences between monogamous and polyandrous individuals when assessed with access to only one male [44]. In contrast, in the present study, reproductive success of single pairs showed remarkable differences between monogamous and polyandrous backgrounds. In particular, our results on standard quality food show that reproductive benefits were most pronounced in crosses between polyandrous females with polyandrous males. In addition, on poor quality food, matings of monogamous females with polyandrous males resulted in improved relative reproductive output. This could also suggest that sex-specific evolution in the **P**- *vs.* the **M**-regime has selected for different reproductive traits or loss of traits leading to different reproduction-survival trade-offs in response to food quality [53]. In life-history theory such trade-offs are expected to depend on physiological and ecological conditions [54,55]. Facing poor conditions, monogamous individuals might be able to save energy by investing less into reproduction, as has been shown in *Drosophila melanogaster* following stress exposures [56]. In contrast, polyandrous males, which have evolved under constant competition, are expected to opt for maximal early reproduction, although this might increase the chance of disease or even death [54]. Nevertheless, this does not seem to be the case in our study, as the relative differences between **M** × **M** and **P** × **P** crosses are similar in both environments. The key difference when comparing across the two environments relates to the cross between **M**-females and **P**-males, where females seem to profit from mating with these males in the stressful (but not benign standard) environment. This suggests that in the stressful environment, benefits of sexual selection outweigh costs of sexual conflict incurred by **M**-females mating with **P**-males. Nevertheless, it is not clear what precise underlying mechanisms and traits drive the different costs and benefits for **M**-females paired with **P**-males across contrasting environments. Previous studies have shown that under polyandry males can evolve larger testes or increased sperm numbers [57-60] and in females, monogamy may lead to inferior oviposition rates compared to polyandry [44,61,62], and decrease female resistance to male harm [62].

Our results suggest that polyandrous females are reproductively fitter than monogamous females (see also [45]), which might be an indication that they invest more energy into producing eggs. Using the ancestral *T. castaneum* strain of our selection lines, Sbilordo et al. [47] found that well-fed males (flour with 10% yeast) provided the female with significantly more sperm for egg fertilization than starved males. Thus, in combination, this might suggest that nutritional effects on polyandrous males and polyandrous females and their elevated reproductive investment might be additive, thus resulting in the highest reproductive success found overall. Hellriegel and Blanckenhorn [63] investigated male sexual traits in *Scatophaga stercoraria* and showed that male investment in reproduction was not very sensitive to effects of food quality, indicating that certain sexually selected traits might not be very plastic in response to variation in nutrition. However, compared to this naturally polygamous species, in our **P**-regime sexual selection pressure on both males and females was potentially much higher due to the constant 1:5 female to male sex ratio. To gain broader insights into reproduction-survival trade-offs it would be fruitful to assess further measures of reproductive success. This could include the number of offspring produced during a time window matching the selection period of seven to ten days, as well as reproductive success over their full natural life span (NB *T. castaneum* are long-lived, frequently living more than a year in the laboratory, see [64,65]).

## Conclusions

We have experimentally shown here that fitness costs and benefits of sexually selected adaptations can shift due to environmental effects. In their standard food quality environment, to which the beetles were well adapted, costs of sexual conflict appear to be stronger, with **M**-females potentially harmed by mating with **P**-males. In contrast, in the novel and stressful low food quality environment, benefits of sexual selection seem to be predominant, as **M**-females profit from mating with **P**-males. Previous work on *Drosophila melanogaster*[66] suggests that in well-adapted populations, detrimental effects associated with sexual conflict may outweigh beneficial effects of sexual selection. It is entirely feasible that the converse would be true in environments to which populations are poorly adapted. Regardless of the precise underlying mechanisms, which might have caused the observed differences in reproductive success, our findings have implications for future studies investigating consequences of environmental change. Our results confirm that low population sizes associated with mating with fewer individuals in combination with poor environmental conditions could lead to a vicious circle with population productivity continuously declining and extinction risk increasing. In order to improve our current understanding of biodiversity declines and to develop management strategies following severe habitat changes, it would be fruitful to investigate the role of mating systems and their ecological sensitivity in greater detail. As highlighted with regard to mating systems in plants [67], our findings suggest that in animals, breeding systems and their consequences for sexual selection should not be neglected.

## Abbreviations

M: Monogamous; P: Polyandrous; RS: Reproductive success.

## Competing interests

The authors declare that they have no competing interests.

## Authors’ contributions

ŁM, OYM and MJGG initiated and maintained the experimental evolution lines used in this work. MD and OYM designed and performed the experiments described, and VMG, MD and OYM analyzed the data. VMG and OYM wrote the paper, and all authors discussed and approved the final manuscript.
